# Tunable Capillary Wicking of 316L Stainless-Steel Hierarchical Microgrooves Fabricated by Master Oscillator Power Amplifier Nanosecond Laser Texturing

**DOI:** 10.3390/ma19214619

**Published:** 2026-10-30

**Authors:** Shiwei Zhang, Musen Lin, Weijie Zhang, Yu Wang, Fangqiong Luo, Huarong Qiu, Yanguang Zhou, Gong Chen, Yong Tang

**Affiliations:** 1 Intelligent Manufacturing Engineering Laboratory of Functional Structure and Device in Guangdong, School of Mechanical and Automotive Engineering, South China University of Technology, Guangzhou 510640, China; swzhang@scut.edu.cn (S.Z.); musenlin020331@gmail.com (M.L.); 202521003016@mail.scut.edu.cn (W.Z.); 202330741971@mail.scut.edu.cn (Y.W.); menfqluo@mail.scut.edu.cn (F.L.); gongchen@scut.edu.cn (G.C.); 2 Department of Mechanical and Aerospace Engineering, Hong Kong University of Science and Technology, Clear Water Bay, Kowloon, Hong Kong, China; maeygzhou@ust.hk; 3 School of Mechanical and Control Engineering, Shenzhen University, Shenzhen 518000, China; ytang@scut.edu.cn

**Keywords:** MOPA nanosecond laser texturing, hierarchical microgroove wicks, recast ridges, capillary transport, ultrathin vapor chambers

## Abstract

Laser-induced recast ridges can create auxiliary capillary spaces but may also restrict the main liquid-transport channels. How these competing structural effects influence capillary transport remains insufficiently understood. Capillary pressure depends on surface tension, wettability, and meniscus curvature, whereas viscous resistance depends on fluid viscosity, the available flow cross-section, and channel connectivity. Hierarchical microgroove wicks were fabricated on 316L stainless steel using master oscillator power amplifier (MOPA) nanosecond laser texturing, with scanning passes, groove spacing, and pulse width varied systematically. Scanning electron microscopy and three-dimensional surface profilometry were combined with apparent water contact-angle and capillary-rise measurements to relate structural development to liquid transport. At a groove spacing of 0.1 mm and a pulse width of 200 ns, increasing the scanning passes from 15 to 25 reduced the main-microgroove width from 60 to 55 μm and was accompanied by a decrease in the capillary factor from 30.859 to 29.024 mm·s^−0.5^. The observations indicate that the presence of secondary microgrooves alone is insufficient for improved wicking. Open main–secondary connections are associated with enhanced capillary transport, while excessive recast accumulation restricts channel openings and may increase viscous resistance. These findings provide an experimental basis for controlling recast geometry and channel connectivity in directly textured stainless-steel wicks for potential use in ultrathin vapor chambers.

## 1. Introduction

Capillary wicking drives liquid transport in porous media [1], microgrooves [2], and two-phase devices such as heat pipes [3,4] and vapor chambers [5]. Governed by capillary pressure, wettability, channel geometry, and viscous resistance, it returns condensate to the evaporator and influences the capillary limit, dryout resistance, and operational stability. Increasing power density and electronic-device miniaturization [6] demand thin, efficient wicks within restricted internal space [7]. Conventional designs include sintered powders [8], metal meshes [9], regular microgrooves [10], and composite wicks [3,11]. Powder and mesh wicks generally require separate layers that occupy vapor-core space, whereas directly machined microgrooves offer integrated, relatively low-resistance flow paths. However, smaller grooves increase capillary pressure but may raise viscous resistance, while larger grooves favor flow at lower capillary pressure. Multiscale wicks combine these advantages to balance pumping and transport [12]. Here, the term “multiscale” refers to capillary structures combining features with distinguishable characteristic dimensions, such as relatively large liquid-transport grooves and finer pores or protrusions.

Laser processing offers a promising approach for the in situ fabrication of hierarchical wick structures. Compared with mechanical machining [13], chemical etching [14], and the assembly of separate wick layers [15], laser processing is a non-contact technique that enables flexible tool-path design and the tailoring of local structural features [16]. Consequently, microgrooves and micro/nanostructures can be fabricated directly on the shell surface, thereby reducing the internal space occupied by additional wick layers. In recent years, nanosecond lasers have been employed to fabricate parallel microgrooves, dual-scale porous/grooved structures, gradient microgrooves, and multiscale capillary structures [17]. Surface roughness describes height variations evaluated at specified spatial scales, whereas texture geometry refers here to groove width, depth, spacing, recast-ridge shape, and channel interconnections. Roughness can modify apparent wettability and local liquid uptake, but may also promote contact-line pinning [2,18]. Its effect depends on the wetting state and surface chemistry. Groove geometry influences meniscus curvature, available flow space, and the balance between capillary driving and viscous resistance. Consequently, greater roughness or faster surface spreading does not necessarily imply better sustained capillary transport. For example, laser-fabricated multiscale microgroove wicks may consist of micrometer-scale grooves, nanopores, and resolidified material features, and their capillary performance is strongly influenced by parameters such as scan spacing, laser power, repetition rate, and scanning speed [19]. These observations indicate that the performance of laser-fabricated wicks is governed not only by the regular grooves themselves but also by additional laser-induced surface features that contribute to liquid transport.

**Figure 1:**
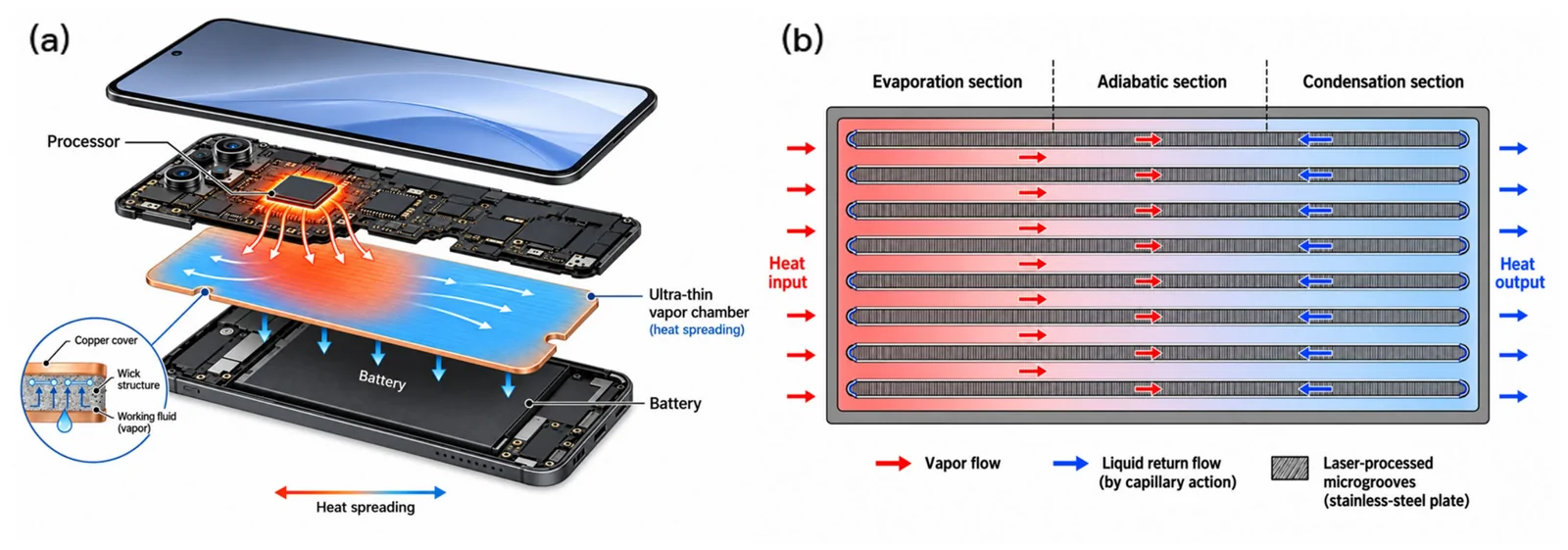
(Figure 1 was prepared with assistance from ChatGPT (GPT-5.1, OpenAI) for graphical ren-dering and visual refinement.) (**a**) Application of an ultrathin vapor chamber in electronic devices; (**b**) operating principle of the ultrathin vapor chamber.

However, the pronounced thermal interaction between nanosecond laser pulses and metallic materials can induce local melting, material migration, ejection, and rapid resolidification, producing recast layers, recast ridges, and redeposited structures along the groove edges [20]. In conventional laser micromachining, these features are generally regarded as defects that degrade dimensional accuracy and machining quality. Excessive recast material may also narrow the groove opening and reduce the effective flow cross-section [21,22]. By contrast, previous studies on capillary transport have shown that laser-induced protrusions, surface roughness, and multiscale structures can enhance surface wettability and generate additional capillary action under appropriate conditions [23]. Therefore, recast structures in laser-fabricated wicks should not be regarded solely as machining defects; their geometry and the local capillary spaces formed between them and the main microgrooves also merit attention. During repeated scanning, resolidified material accumulates along the groove edges and can form longitudinal secondary microgrooves between the recast ridges and the sidewalls of the main microgrooves. When appropriately developed and interconnected, these secondary microgrooves provide additional capillary driving force while the main microgrooves retain relatively large liquid-flow spaces. Excessive recast accumulation, however, can restrict the effective flow cross-section of the main microgrooves and increase liquid-flow resistance. The structural conditions under which secondary microgrooves enhance or hinder capillary transport remain unclear, particularly when the number of scanning passes, groove spacing, and pulse width are varied. To address this issue, hierarchical microgroove structures were fabricated on 316L stainless steel using MOPA nanosecond-laser texturing, and the effects of these processing parameters on structural evolution and capillary transport were systematically investigated. In this study, “hierarchical microgrooves” refers to the structural arrangement of main microgrooves, recast ridges, and recast-ridge-induced secondary microgrooves. Both groove types are micrometer-scale features, and the term “hierarchical” describes their structural relationship rather than distinct characteristic length scales. SEM, 3D surface profilometry, contact-angle and complete-wetting-time measurements, and dynamic capillary-rise tests were used to relate the processing parameters and hierarchical structures to capillary performance. This study links recast-ridge morphology and main–secondary microgroove connectivity to capillary transport, clarifying the dual role of laser-induced recast structures in creating auxiliary capillary pathways and restricting channel connections. These findings provide an experimental basis for controlling recast structures, designing hierarchical microgroove wicks, and developing integrated shell–wick structures for ultrathin stainless-steel vapor chambers. The operating principle of the vapor chamber is illustrated in Figure 1.

## 2. Experimental Section

### 2.1. Materials and Sample Preparation

To minimize substrate bending and warping caused by thermal effects during laser processing and their influence on subsequent measurements, 1 mm-thick 316L stainless-steel plates were used as substrates. The plates were purchased from Taizhou Xuance Metal Products Co., Ltd., Taizhou, China, and their chemical composition is listed in Table 1. Before processing, the samples were ultrasonically cleaned sequentially in absolute ethanol and deionized water and then dried naturally at room temperature. During laser processing, each sample was placed horizontally on the processing stage, and the stage height was adjusted so that the target surface was located at the laser focal plane. Stainless-steel pressing blocks were placed around the sample to further suppress thermal warping and defocusing during repeated scanning. A fan continuously supplied airflow near the processing region to remove particles and debris generated by laser ablation and molten-material ejection, thereby reducing redeposition on the processed surface. Parallel linear scanning was then performed along the prescribed paths, and the number of scanning passes, groove spacing, and pulse width were varied to regulate the formation of the main microgrooves, recast ridges, and secondary microgrooves. Substrates of the same thickness were used for the subsequent morphology, wettability, and capillary-rise tests to ensure consistency among the different measurements and to improve the structural stability of the 150 mm-long strip specimens during capillary rise. The surface morphology and geometric dimensions after laser processing were characterized by scanning electron microscopy (SEM; SU8600, Hitachi High-Tech Corporation, Tokyo, Japan) and 3D surface profilometry(RTEC Up S-Dual, Rtec-Instruments Inc., San Jose, CA, USA). Wettability was evaluated using the contact angle and complete wetting time, and capillary transport was characterized by dynamic capillary-rise experiments.

### 2.2. Formation of Laser-Induced Secondary Microgrooves

**Figure 2:**
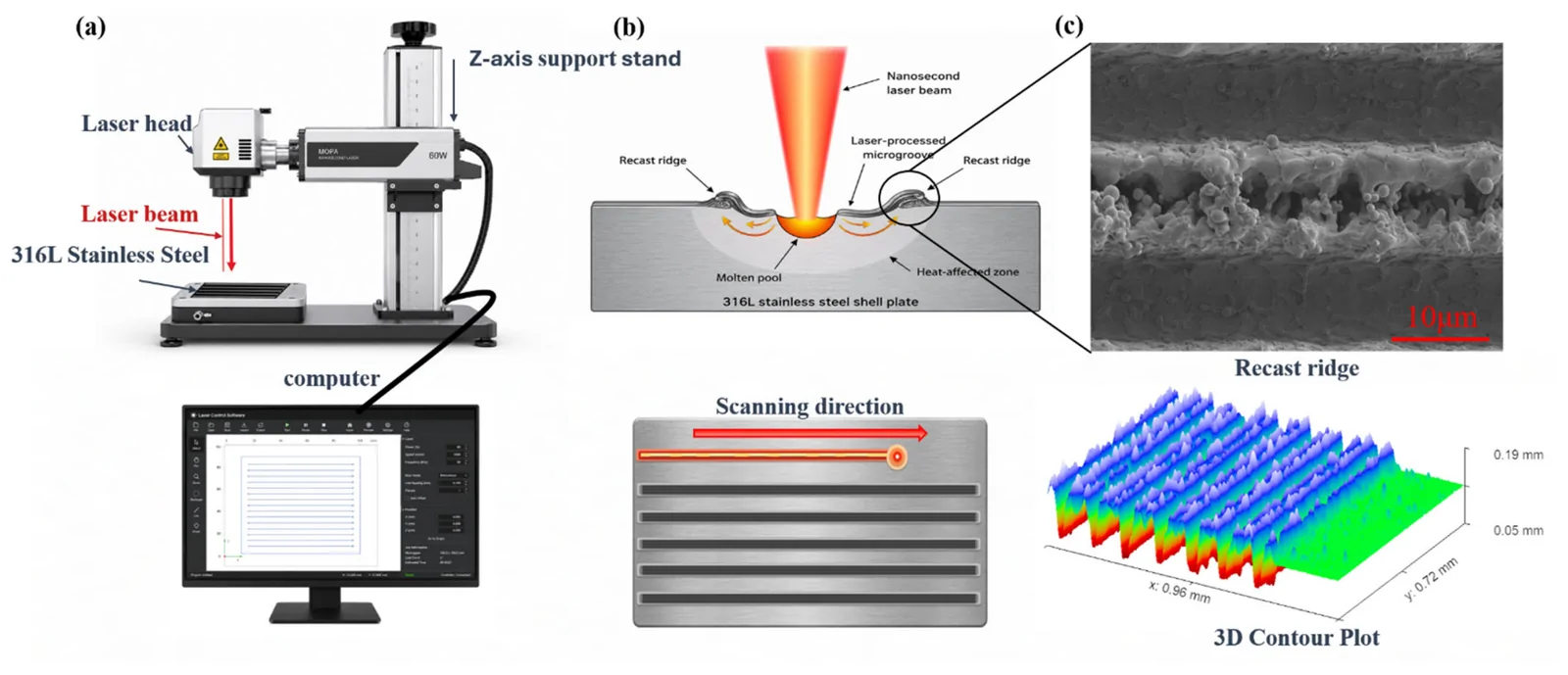
(ChatGPT (GPT-5.1, OpenAI) was used only for rendering the schematic in (**b**)) MOPA nanosecond laser texturing of 316L stainless steel: (**a**) laser processing system; (**b**) schematic illustration of laser processing on the 316L stainless steel surface; and (**c**) morphological characterization of the laser-textured surface.

Figure 2 shows the MOPA nanosecond-laser processing system. An LS-FAST-60W MOPA nanosecond fiber laser (Dongguan Lansu Laser Equipment Co., Ltd., China) was used to machine microgrooves on 316L stainless steel. The laser wavelength was 1064 nm, the spot diameter was approximately 60 μm, the rated power was 60 W, and the repetition rate was fixed at 50 kHz. During processing, the laser power and scanning speed were fixed at 50% of the rated power and 1000 mm/s, respectively, and parallel linear scanning was employed. Taking advantage of the adjustable pulse parameters of the MOPA laser, the number of scanning passes, groove spacing, and pulse width were selected as the principal control parameters, as listed in Table 2, to systematically investigate the formation and evolution of the main microgrooves, recast ridges, and recast-ridge-induced secondary microgrooves under different laser-processing conditions. For each parameter series, the five processing conditions were applied to separate regions of the same 316L stainless-steel plate. This fabrication procedure was repeated on multiple plates, and corresponding regions processed under the same condition were used for subsequent characterization and capillary-rise tests.

### 2.3. Surface Morphology and Wettability

The surface morphology and wettability of the laser-textured 316L stainless-steel samples were characterized using SEM, a 3D surface profilometer, and a contact-angle goniometer. First, SEM (SU8600, Hitachi, Japan) was used to examine the microgroove arrays, recast-ridge structures, molten/recast particles, and secondary microgrooves formed under different laser-processing conditions, allowing the effects of scanning passes, groove spacing, and pulse width on the evolution of the surface morphology to be assessed. EDS results for the untreated reference and the preferred-condition laser-textured specimen are provided in the Supplementary Materials (Figures S1–S3 and Table S1). A 3D surface profilometer (RTEC Up S-Dual, RTEC, Rtec-Instruments Inc., San Jose, CA, USA) equipped with a 20× objective was then used to obtain surface-height maps and cross-sectional profiles of the laser-textured regions. The field of view was 0.958 mm × 0.7185 mm, with a sampling grid of 1280 × 960 data points, corresponding to a lateral sampling interval of approximately 0.748 μm in both the *x*- and *y*-directions. The main microgroove depth (defined as the vertical distance from the apex of the recast ridge to the groove bottom), the depth from the original reference surface to the main-groove bottom, the main microgroove width, and the depth and width of the secondary microgrooves were extracted from the profiles. Finally, surface wettability was measured using an optical contact-angle goniometer (OCA 50, DataPhysics Instruments GmbH, Filderstadt, Germany) with deionized water as the test liquid. Deionized water was selected because it is commonly used as a working fluid in vapor chambers, making the wettability assessment more relevant to the intended application of the present wick structures. Multiple measurements were performed at different positions on each sample, and the average value was used as the representative contact angle for evaluating the influence of the laser-induced surface structure on wettability.

### 2.4. Capillary Rise Test and Data Analysis

**Figure 3:**
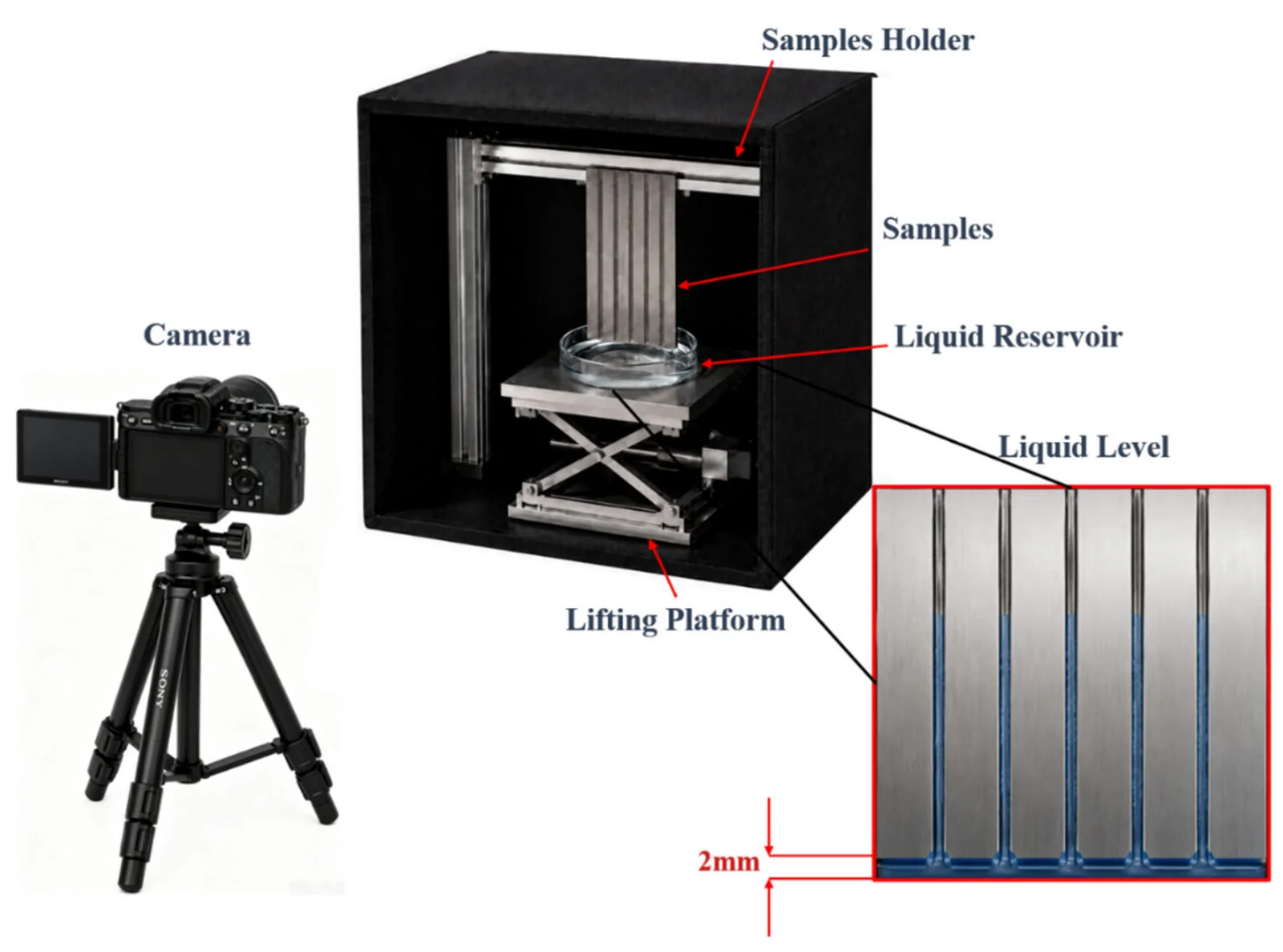
(ChatGPT (GPT-5.1, OpenAI) was used only for rendering the schematic in Figure 3.) Schematic diagram of the capillary rise test setup.

Figure 3 shows the capillary-rise test setup for the laser-textured 316L stainless-steel surfaces. A high-speed camera was used to record the dynamic rise in liquid along the main microgrooves and recast-ridge-induced secondary microgrooves at a resolution of 2K and a frame rate of 60 Hz. Deionized water was used as the working fluid, and all capillary-rise tests were conducted at 25 °C. Before each test, the container filled with deionized water and the specimen were placed in a black enclosure for 1 h, allowing evaporation to establish an atmosphere close to saturated water vapor. This procedure minimized changes in the reservoir liquid level and errors in identifying the liquid front caused by evaporation during capillary rise, thereby improving measurement reliability. During testing, the sample was fixed vertically with the liquid-rise direction parallel to the microgrooves. The reservoir level was adjusted using a lifting platform so that the lower end of the sample was immersed by approximately 2–3 mm, while the camera continuously recorded the motion of the liquid front. After the experiment, the liquid-front height was extracted using Tracker software (version 6.2.0). Data were recorded every 0.1 s over 0–2 s, every 0.5 s over 2–10 s, every 1 s over 10–20 s, every 5 s over 20–60 s, and every 10 s thereafter until the liquid front reached the upper limit of the observation region or remained essentially stable.

The capillary structure formed on laser-textured 316L stainless steel consists of the main microgrooves, recast ridges, and recast-ridge-induced secondary microgrooves, resulting in an open hierarchical liquid-transport network. Consequently, a single geometric radius used for a regular capillary tube or the intrinsic permeability used for a homogeneous porous medium cannot directly characterize the overall capillary performance of this hierarchical structure. An equivalent-parameter model based on the Young–Laplace relation [24] and Lucas–Washburn theory [25] was therefore adopted. Capillary-rise tests were performed for the CJ, CJS, and CJPW series, and the experimentally obtained capillary factor W was used as the primary metric for evaluating capillary transport performance.

During capillary rise, the liquid is primarily driven by capillary pressure and opposed by viscous and hydrostatic pressure drops. The pressure balance can be written as:

(1) $$\Delta P_{\mathrm{cap}}=\Delta P_{\mu }+\Delta P_{g}$$

where $\Delta P_{cap}$, $\Delta P_{\mu }$ and $\Delta P_{g}$ denote the capillary pressure, viscous pressure drop, and gravitational pressure drop, respectively.

For the complex open hierarchical microgrooves considered here, the combined capillary action generated by the main microgrooves, secondary microgrooves, and their interconnected spaces is expressed in an equivalent form as:

(2) $$\Delta P_{cap}=\frac{2\sigma \cos\theta }{R_{eff}}$$

where σ is the surface tension of the working fluid, θ is the contact angle between the liquid and the laser-textured surface, and $R_{eff}$ is the effective capillary radius used to represent the overall capillary length scale of the hierarchical microgrooves.

The liquid-flow resistance in the microgroove structure can be described using Darcy’s law [26]:

(3) $$\Delta P_{\mu }=\frac{\mu h}{K_{eff}}\frac{dh}{dt}$$

where μ is the dynamic viscosity of the working fluid, h is the liquid-rise height, dh/dt is the instantaneous liquid-rise velocity, and $K_{eff}$ is an equivalent transport parameter representing the overall liquid-transport capability of the hierarchical microgrooves. Because the present structure consists of open microgrooves rather than a homogeneous porous medium, $K_{eff}$ is used only as an equivalent parameter to describe the viscous flow resistance generated by the structure and is not equivalent to the intrinsic permeability obtained from a standard porous-medium measurement.

The gravitational pressure drop associated with the liquid column can be expressed as

(4) $$\Delta P_{g}=\mathrm{\rho gh}$$

where ρ is the density of the working fluid and g is the gravitational acceleration.

Substituting Equations (2)–(4) into Equation (1) gives the equivalent governing relation for capillary rise in the hierarchical microgrooves:

(5) $$\frac{2\sigma \cos\theta }{R_{eff}}=\frac{\mu h}{K_{eff}}\frac{dh}{dt}+\mathrm{\rho gh}$$

Equation (5) shows that, during the initial stage of capillary rise, the liquid-column height is small and the gravitational pressure drop is weak relative to the capillary pressure; the gravitational term can therefore be neglected approximately. Equation (5) can then be simplified and integrated to give

(6) $$h=\sqrt{\frac{4\sigma K_{eff}\cos\theta }{\mu R_{eff}}}\sqrt{t}=W\sqrt{t}$$

where W is the capillary factor. For a given wick structure, working fluid, and testing condition, W can be regarded as a constant. Therefore, during the initial stage of capillary rise, h is approximately linear with $\sqrt{t}$, and W can be obtained as the slope of a linear fit of h versus $\sqrt{t}$.

The average liquid-rise velocity, $V_{avg}$ is defined as

(7) $$V_{\mathrm{avg}}=\frac{h}{t}$$

The dynamic capillary-rise process on the laser-textured 316L stainless-steel microgrooves was recorded using a camera, and the liquid-front height h was extracted at different times using the image scale; the measurement uncertainty was less than 2%. The experimental time t was recorded by the image-acquisition system. The capillary factor W was obtained by linearly fitting h versus $\sqrt{t}$ during the initial stage of capillary rise, and the average capillary-rise velocity $V_{avg}$ was calculated from the change in liquid-front height over the specified time interval. Based on standard uncertainty-propagation analysis and considering the measurement errors in rise height and time, the uncertainties in W and $V_{avg}$ were both controlled within 2%.

Previous studies have investigated capillary suction in textured microcavities and capillary filling in cylindrical tubes [27,28]. In this study, the Lucas–Washburn relation was used to characterize the initial capillary-rise behavior through the capillary factor W obtained by fitting the experimental data. The measured groove dimensions provided a geometrical basis for interpreting the differences in capillary performance among processing conditions. However, the effective parameters *R_eff_* and *K_eff_* were not independently determined from these dimensions. Therefore, the present analysis provides an experimental comparison of capillary transport performance rather than an independent prediction of capillary-rise height from the groove geometry.

## 3. Results and Discussion

### 3.1. Surface Morphology Characterization

**Figure 4:**
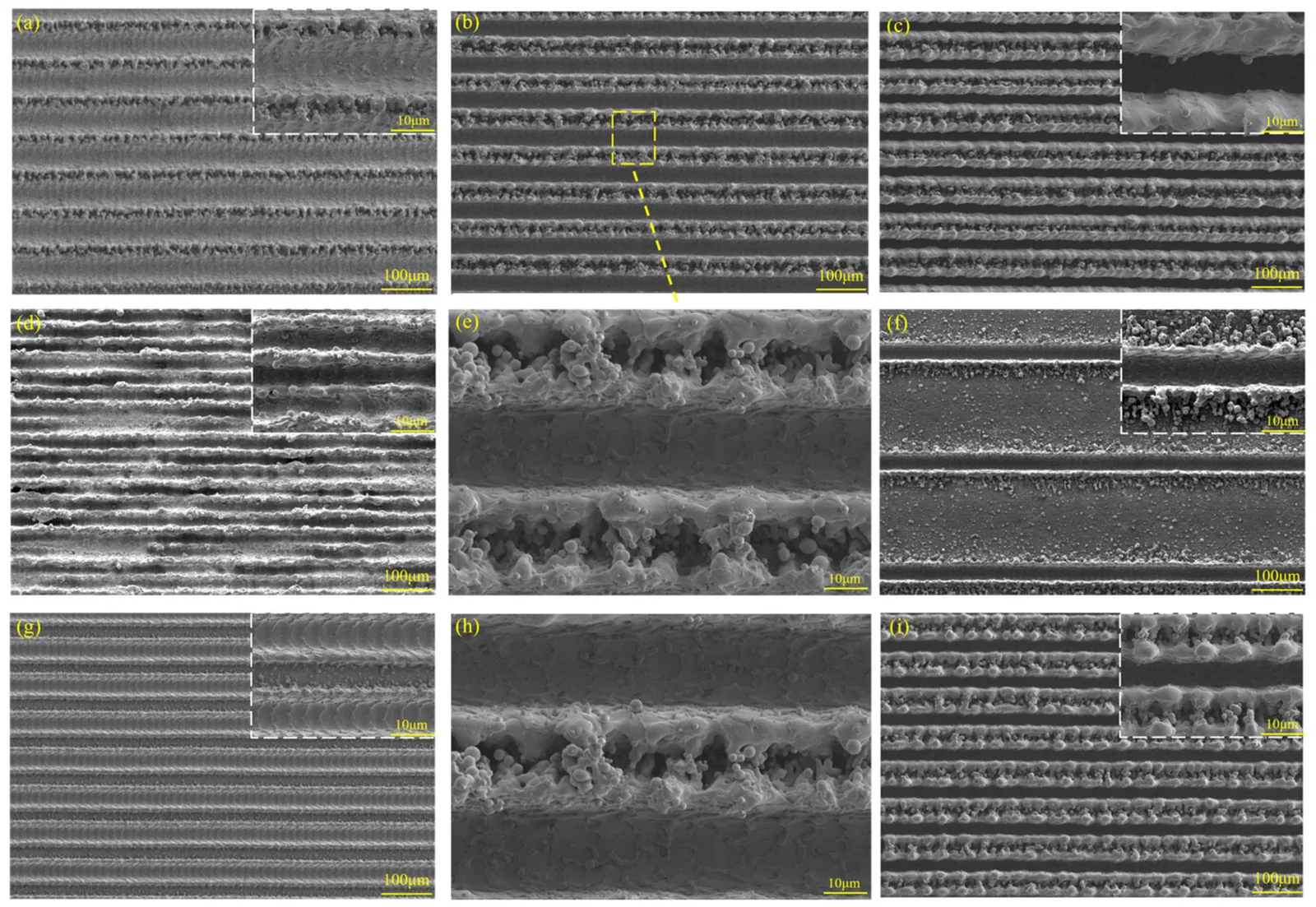
SEM images of laser-textured 316L stainless steel surfaces under different processing conditions: (**a**) CJ5; (**b**) CJ15; (**c**) CJ25; (**d**) CJS0.05; (**e**) CJS0.1; (**f**) CJS0.2; (**g**) CJPW50; (**h**) CJPW200; and (**i**) CJPW400. CJ15, CJS0.1, and CJPW200 correspond to the same processing condition.

As summarized in Table 3, the geometrical parameters are reported as mean values obtained from 3D surface profilometry measurements at multiple locations within each textured region. Across the laser-textured samples, the mean widths and depths of the main microgrooves ranged from 48 to 85 μm and from 67.4 to 138.7 μm, respectively. The main-microgroove depth was defined as the vertical distance from the recast-ridge apex to the groove bottom. The corresponding mean depths measured from the original reference surface ranged from 41.8 to 79.9 μm. For samples with identifiable open secondary microgrooves, their mean widths and depths ranged from 43 to 64.1 μm and from 20 to 54.6 μm, respectively. Both the main and secondary microgrooves were micrometer-scale features. To clarify the evolution of the hierarchical microgroove structure, three representative samples showing distinct morphological changes were selected from each parameter series for SEM examination, as presented in Figure 4. Main microgrooves were observed in all selected samples, whereas the development of the recast ridges and secondary microgrooves varied with the processing parameters. As shown in Figure 4a–c, increasing the number of scanning passes deepened the main microgrooves and promoted recast accumulation along their edges. CJ5 exhibited low recast ridges and only incipient secondary features. At 15 passes, distinct secondary microgrooves formed between the recast ridges and the main-groove sidewalls. At 25 passes, further accumulation produced higher recast ridges and narrowed the upper region of the main microgrooves. Figure 4d–f show the representative changes caused by groove spacing. At 0.05 mm, strong overlap between adjacent laser-affected regions caused neighboring recast structures to merge, with no distinct open secondary microgrooves discernible in the final surface morphology. At 0.1 mm, the main microgrooves, recast ridges, and secondary microgrooves were clearly distinguishable. When the spacing increased to 0.2 mm, the interaction between adjacent scanning paths weakened, and secondary microgrooves were no longer evident. The pulse-width series showed a different morphological evolution from that caused by scanning passes (Figure 4g–i). At short pulse widths, the limited interaction time restricted heat diffusion and molten-material migration, and no identifiable open secondary microgrooves were observed in the final surface morphology. This differs from the low-scanning-pass condition, in which incipient secondary features were already present. At 200 ns, sufficient melt displacement and resolidification produced distinct secondary microgrooves. Further increasing the pulse width enlarged the recast ridges and secondary microgrooves but reduced the opening of the main microgrooves.

**Figure 5:**
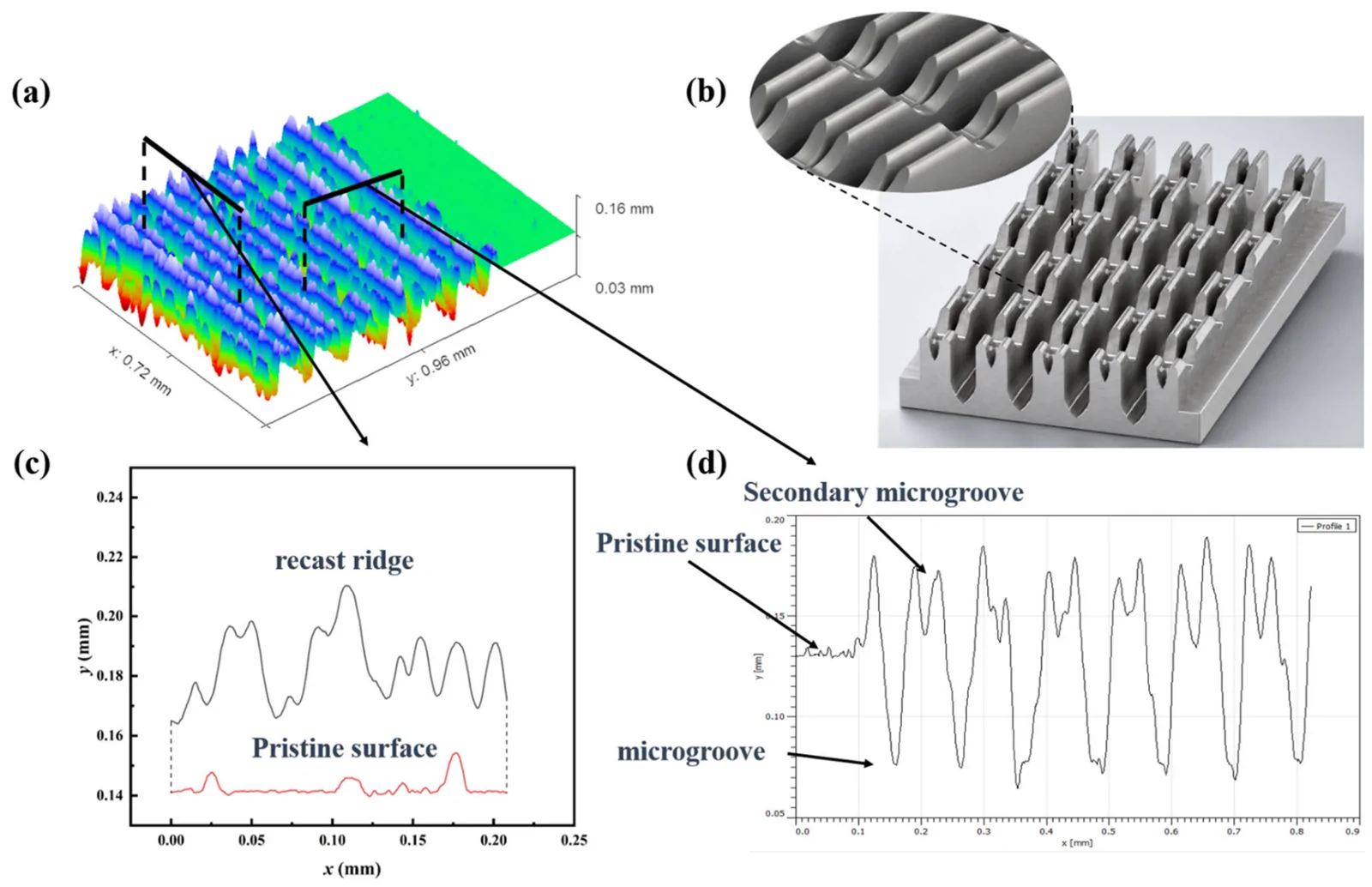
(**a**) 3D surface topography of the sample processed with 15 scanning passes, a groove spacing of 0.1 mm, and a pulse width of 200 ns; (**b**) schematic of its interconnected main–secondary microgroove structure; (**c**) corresponding *x*-direction surface profile; and (**d**) corresponding *y*-direction surface profile.

**Figure 6:**
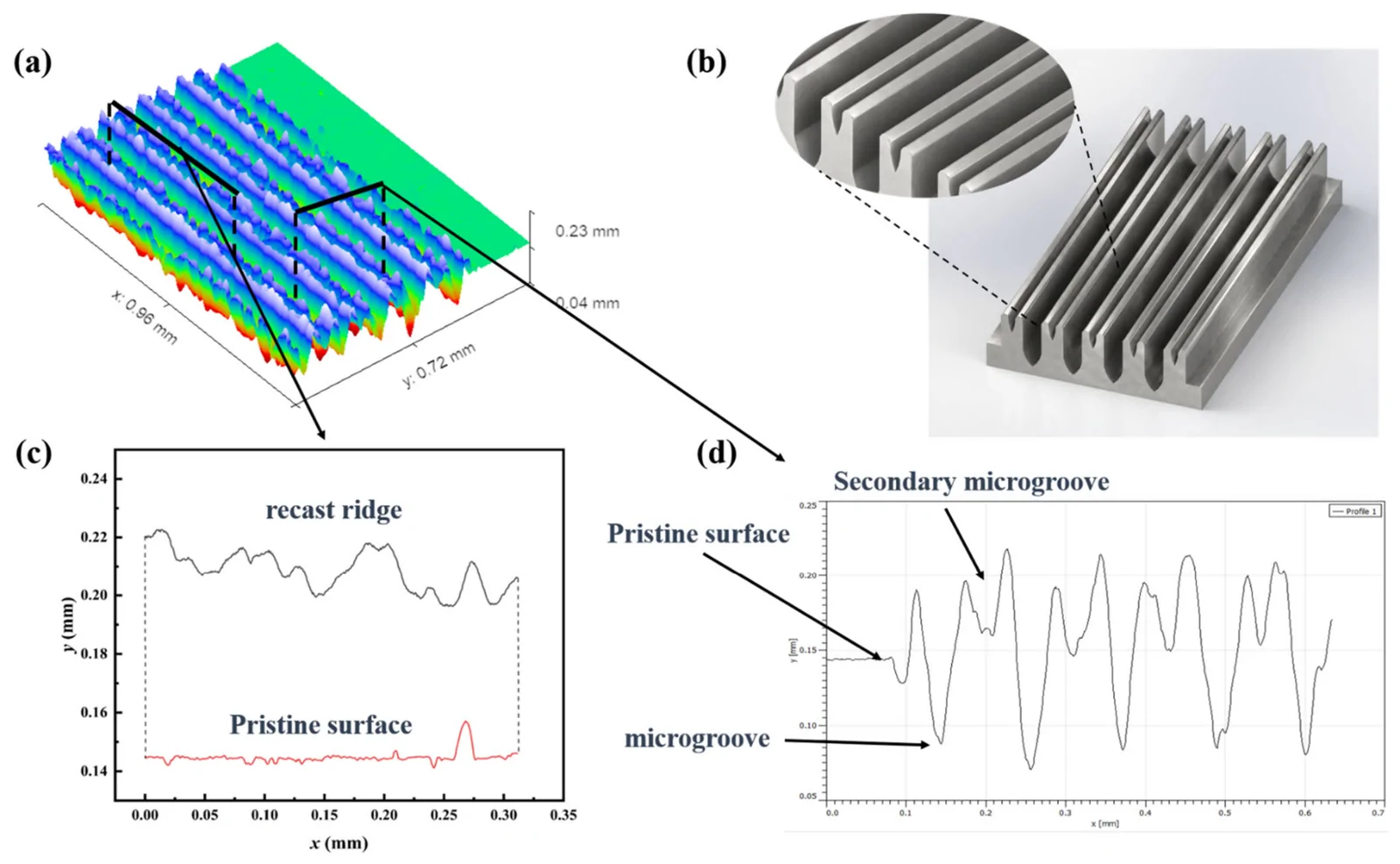
(**a**) 3D surface topography of the sample processed with 25 scanning passes, a groove spacing of 0.1 mm, and a pulse width of 200 ns; (**b**) schematic of its continuous wall-like recast-ridge structure; (**c**) corresponding *x*-direction surface profile; and (**d**) corresponding *y*-direction surface profile.

The surface profiles and 3D topographies in Figure 5 and Figure 6 further clarify the effect of recast-ridge morphology on the connection between the main and secondary microgrooves. Because the material accumulation caused by increasing the number of scanning passes was similar to that observed at longer pulse widths, CJ25 was selected to represent the overdeveloped recast structure and was compared with CJ15. Both samples were processed at a groove spacing of 0.1 mm and a pulse width of 200 ns. The x-direction profile of CJ15 in Figure 5c shows a relatively small recast-ridge height above the pristine surface. Local depressions along the ridge formed basin-like openings that connected the secondary microgrooves to the main microgrooves, consistent with the 3D topography and schematic illustration in Figure 5a,b. By contrast, Figure 6c shows that the recast ridges of CJ25 were considerably higher. Repeated scanning caused substantial recast material to accumulate and build upward along the groove edges, producing high, continuous wall-like ridges without the distinct basin-like connections observed in CJ15, as illustrated in Figure 6a,b. The y-direction profiles in Figure 5d and Figure 6d further show that secondary microgrooves were present in both samples, but their connection to the main microgrooves was weakened in CJ25. These observations indicate that secondary microgrooves can effectively assist the main transport channels only when the recast ridges maintain a suitable height and retain basin-like interconnected regions.

#### 3.1.1. Contact Angle and Surface Wettability Characterization

**Figure 7:**
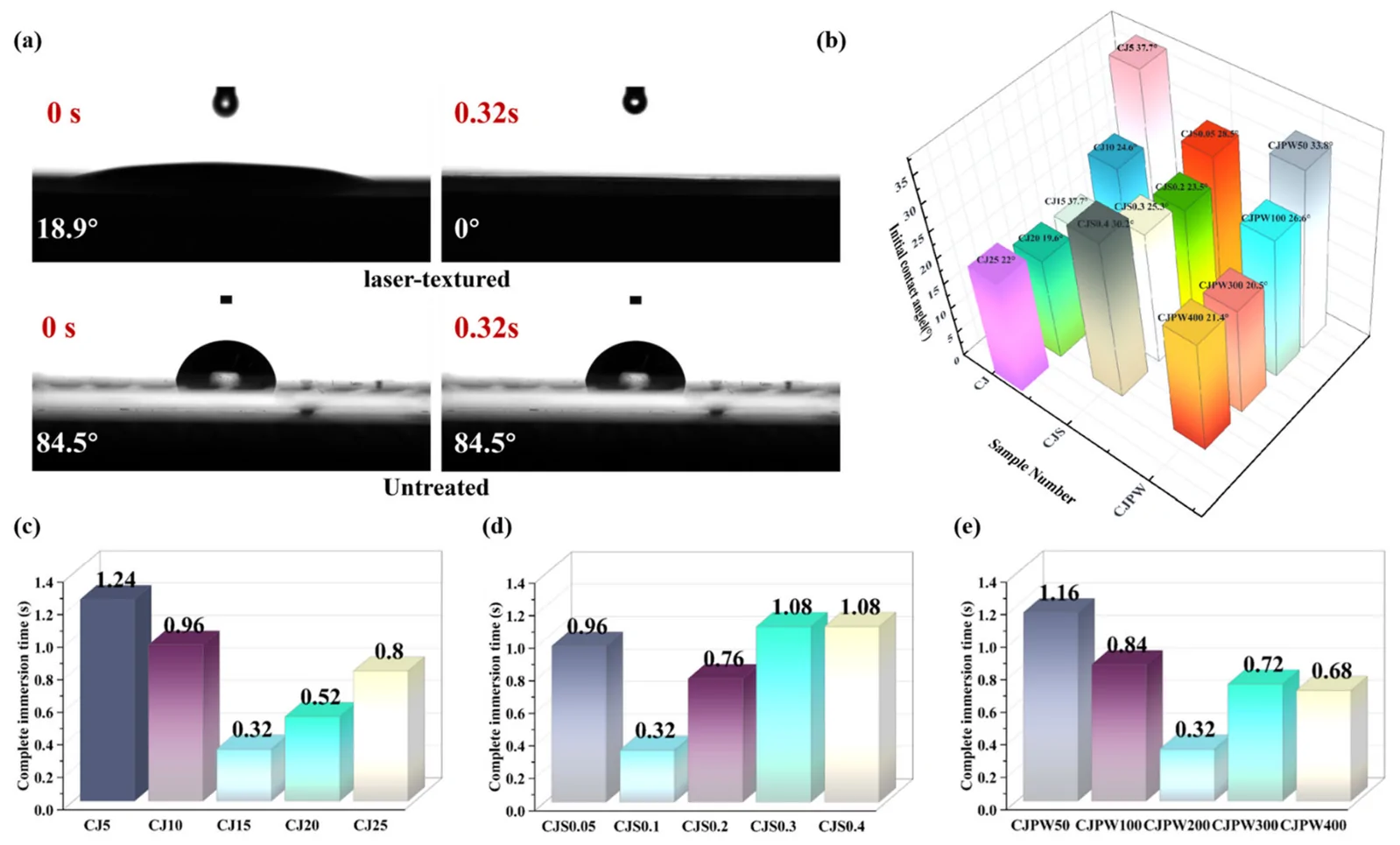
Wettability characterization of 316L stainless steel: (**a**) droplet spreading behavior on the untreated surface and the sample processed under the preferred parameter combination; (**b**) initial contact angles of all samples; and (**c**–**e**) complete wetting times for different scanning passes, groove spacings, and pulse widths, respectively.

To clarify the influence of the hierarchical microgroove morphology described above on surface wetting behavior, Figure 7 compares the contact angle and complete wetting time of the untreated 316L stainless-steel reference surface with those of samples processed under different laser conditions. As shown in Figure 7a, the initial contact angle of the untreated surface was 84.5°, and the droplet retained a pronounced spherical-cap shape, indicating relatively poor wettability. In contrast, under the preferred parameter combination within the investigated range—15 scanning passes, a groove spacing of 0.1 mm, and a pulse width of 200 ns, corresponding to CJ15, CJS0.1, and CJPW200—the initial contact angle decreased to 18.9°. The droplet subsequently spread rapidly and reached a fully wetted state, demonstrating that laser texturing markedly improved the hydrophilicity of 316L stainless steel.

Figure 7b summarizes the initial contact angles of samples processed under different laser conditions. For all three parameter series, the contact angle first decreased and then increased, with the preferred parameter combination giving the lowest value of 18.9°, whereas CJ5 exhibited the highest value of 37.7°. Combined with the SEM and 3D-profile results in the preceding section, these observations indicate that suitable laser parameters produce a well-matched hierarchical structure consisting of main microgrooves, recast ridges, and recast-ridge-induced secondary microgrooves. This hierarchy increases the actual solid–liquid contact area and provides continuous pathways for liquid spreading along the groove direction. Insufficient processing produces shallow main microgrooves and underdeveloped secondary microgrooves, whereas excessive processing causes recast-material accumulation and contraction of the effective channels; both conditions hinder rapid wetting. Figure 7c–e compare the effects of scanning passes, groove spacing, and pulse width on the complete wetting time. CJ15, CJS0.1, and CJPW200 correspond to the same preferred parameter combination and all exhibited a complete wetting time of 0.32 s, substantially shorter than those of the other samples; CJ5 showed the longest complete wetting time of 1.24 s. These results demonstrate that the hierarchical microgrooves formed at 15 scanning passes, a groove spacing of 0.1 mm, and a pulse width of 200 ns provide favorable surface wettability. The contact-angle and complete-wetting-time results also provide a wettability basis for analyzing capillary-rise behavior under different laser-processing conditions in the following section.

#### 3.1.2. Effect of Laser Processing Parameters on Capillary Performance

**Figure 8:**
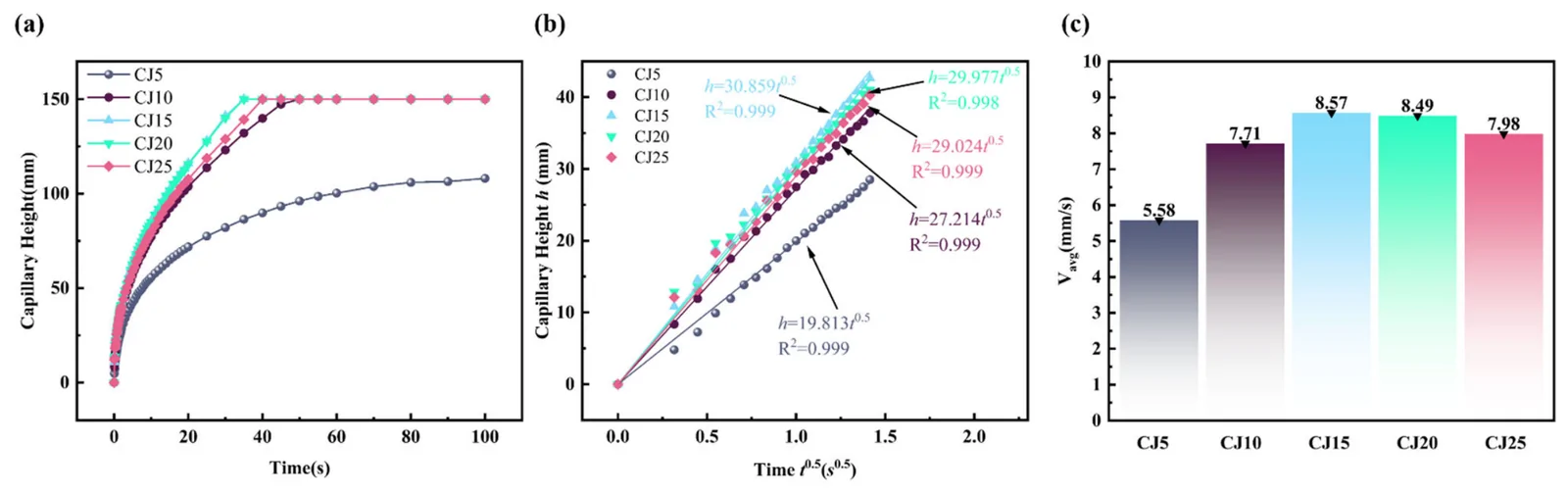
Capillary performance of microgroove wicks fabricated with different numbers of scanning passes: (**a**) capillary rise curves; (**b**) capillary factors determined from the initial 0–2 s; and (**c**) comparison of the average capillary rise velocities within the first 10 s.

Following the contact-angle and complete-wetting-time measurements, dynamic capillary-rise tests were conducted to evaluate liquid transport in the hierarchical microgrooves. All samples showed a rapid initial rise followed by gradual deceleration as gravity and viscous resistance increased with the rising liquid column. Because the maximum observable height was 150 mm, the plateau at this level represents the measurement limit rather than the theoretical equilibrium height. The capillary-rise curves, capillary factors, and average rise velocities in Figure 8 consistently show that capillary performance first improved and then declined with increasing scanning passes. As observed from the SEM images and 3D topographies in the preceding section, secondary microgrooves had begun to form at low scanning passes, but the main microgrooves remained shallow and provided insufficient space for liquid transport. The resulting capillary factors and initial rise velocities were therefore relatively low. At 15 passes, moderate material displacement produced recast ridges of suitable height and basin-like openings that connected the secondary microgrooves to the main microgrooves. This structure combined the transport capacity of the main channels with the additional capillary action of the secondary channels. Consequently, CJ15 exhibited the best performance, reaching the 150 mm measurement limit in approximately 35 s, with a capillary factor of 30.859 mm·s^−0.5^ and an average rise velocity of 8.57 mm·s^−1^ during the first 10 s. Further increasing the scanning passes deepened the main microgrooves but also caused recast material to accumulate around their openings. The resulting high, continuous wall-like ridges restricted the basin-like connections between the main and secondary microgrooves and narrowed the upper flow region of the main microgrooves. Although secondary microgrooves remained present, their auxiliary transport effect was weakened. Accordingly, the capillary factors decreased to 29.977 and 29.024 mm·s^−0.5^ for CJ20 and CJ25, while their average rise velocities declined to 8.49 and 7.98 mm·s^−1^, respectively. These results show that effective capillary enhancement requires not only the presence of secondary microgrooves but also their open connection and structural coordination with the main microgrooves.

**Figure 9:**
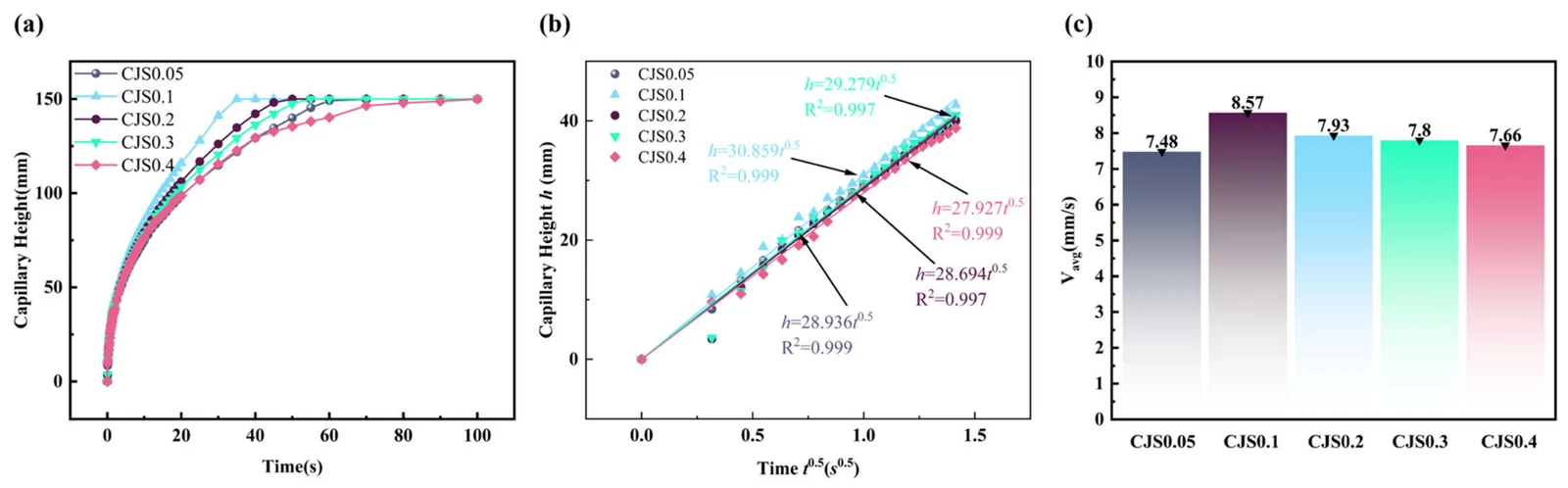
Capillary performance of microgroove wicks fabricated with different groove spacings: (**a**) capillary rise curves; (**b**) capillary factors determined from the initial 0–2 s; and (**c**) comparison of the average capillary rise velocities within the first 10 s.

Figure 9 shows that groove spacing had a non-monotonic effect on capillary performance. At 0.05 mm, extensive overlap between adjacent laser-affected regions caused the recast material to merge, restricting the main-groove flow space and preventing the formation of well-defined secondary microgrooves. CJS0.05 consequently exhibited a capillary factor of 28.936 mm·s^−0.5^ and an average rise velocity of 7.48 mm·s^−1^ during the first 10 s. At 0.1 mm, CJS0.1 corresponded to the same processing condition as CJ15 and therefore possessed the same basin-like secondary microgrooves connected to the main microgrooves. It exhibited the highest capillary factor and average rise velocity in the spacing series. When the spacing increased to 0.2, 0.3, and 0.4 mm, the greater separation weakened the thermal interaction between adjacent scanning paths. The resulting melt displacement and recast-ridge development were insufficient to form distinct secondary microgrooves. Because the processing width was fixed, increasing the spacing also reduced the number of main microgrooves per unit width. Liquid transport therefore occurred primarily through the less densely distributed main microgrooves. The corresponding capillary factors were 28.694, 29.279, and 27.927 mm·s^−0.5^, all lower than that of CJS0.1, while the average rise velocities decreased from 7.93 to 7.80 and 7.66 mm·s^−1^. These results show that increasing the spacing beyond 0.1 mm suppressed secondary-microgroove formation and reduced the overall capillary transport capability.

**Figure 10:**
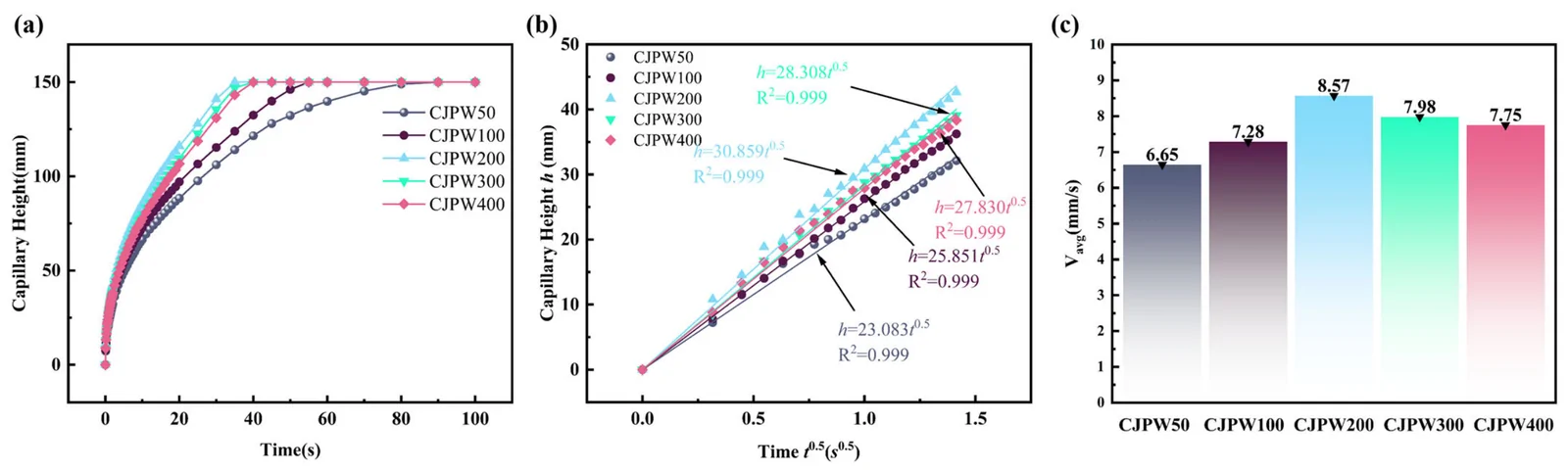
Capillary performance of microgroove wicks fabricated using different laser pulse widths: (**a**) capillary rise curves; (**b**) capillary factors determined from the initial 0–2 s; and (**c**) comparison of the average capillary rise velocities within the first 10 s.

Figure 10 shows that pulse width affected capillary transport by altering the thermal interaction and the resulting microgroove structure. At 50 and 100 ns, the short interaction time limited heat diffusion and molten-material migration, and no identifiable open secondary microgrooves were observed in the final surface morphology. Liquid transport was therefore dominated by the main microgrooves. Nevertheless, the further development of the main microgrooves increased the capillary factor from 23.083 to 25.851 mm·s^−0.5^ and the average rise velocity during the first 10 s from 6.65 to 7.28 mm·s^−1^. At 200 ns, moderate melt migration and accumulation produced recast ridges with basin-like openings, allowing secondary microgrooves to form and connect with the main microgrooves. This hierarchical structure provided the strongest capillary transport, with a capillary factor of 30.859 mm·s^−0.5^ and an average rise velocity of 8.57 mm·s^−1^. When the pulse width was increased to 300 and 400 ns, the prolonged thermal interaction caused excessive recast material to accumulate around the main-groove openings. The resulting high recast ridges narrowed the main channels and weakened their connection with the secondary microgrooves. Accordingly, the capillary factor decreased to 28.308 and 27.830 mm·s^−0.5^, while the average rise velocity declined to 7.98 and 7.75 mm·s^−1^, respectively. Thus, a pulse width of 200 ns provided the most suitable balance between secondary-microgroove formation, channel connectivity, and available flow space.

**Figure 11:**
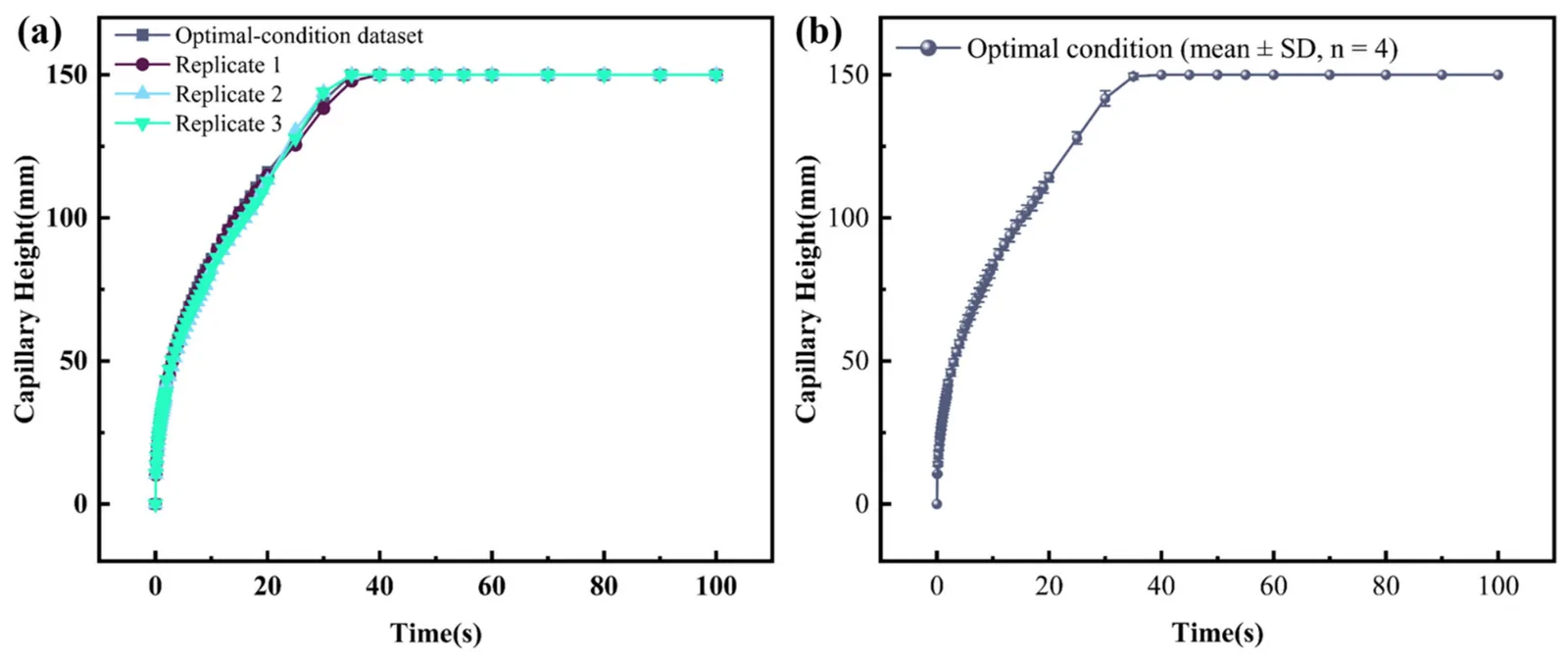
Capillary-rise reproducibility under the preferred condition: (**a**) individual curves from four independently fabricated specimens; (**b**) mean ± SD (n = 4). The 150 mm level represents the measurement limit.

To assess specimen-to-specimen reproducibility, capillary-rise measurements were performed on four independently fabricated specimens under the preferred processing condition. Figure 11a compares the original dataset with three additional replicate datasets, while Figure 11b presents the mean rise height and standard deviation at each measurement time (*n* = 4). The 150 mm level represents the measurement limit.

### 3.2. Comparison with Previous Studies

**Figure 12:**
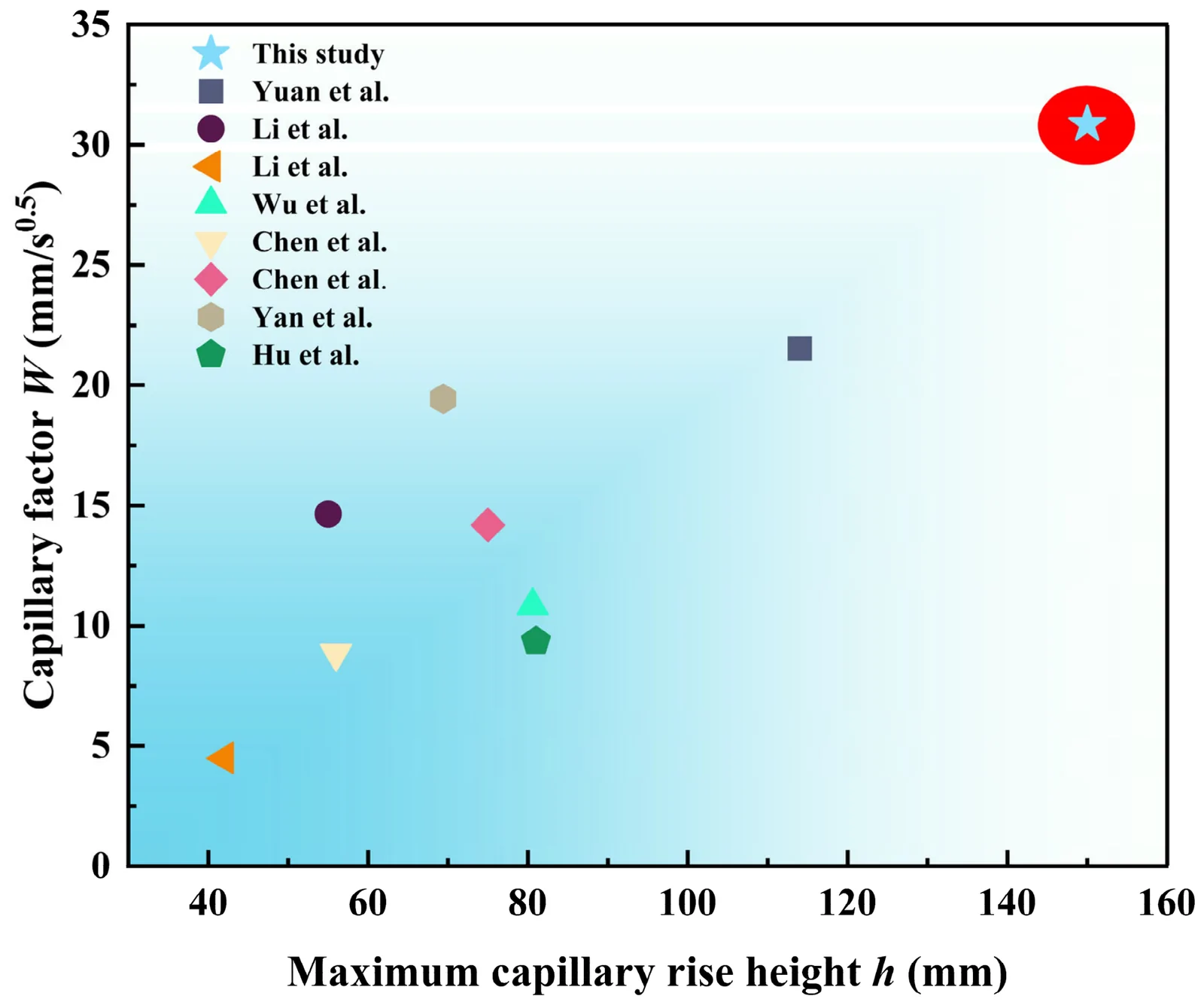
Comparison of comprehensive capillary performance with previously reported studies.

To further evaluate the capillary transport performance of the laser-textured 316L stainless-steel hierarchical microgroove wick, representative wick structures reported in the literature were selected for comparison, as summarized in Figure 12 and Table 4. To provide a consistent comparison, all selected studies used deionized water as the working fluid, and the capillary factor was used as the principal comparative metric.

As summarized in Table 4, powder-sintered wicks [29] exhibit high porosity and strong capillary pumping, with a capillary factor of 14.65 mm·s^−0.5^. Li et al. [30] further investigated a gradient sintered-powder wick fabricated from 316L stainless steel using deionized water as the working fluid. Based on the published capillary-rise data, its capillary factor was recalculated to be approximately 4.5 mm·s^−0.5^. Although sintered-powder wicks provide favorable capillary performance, their fabrication generally involves powder preparation, forming, and sintering processes, which increase manufacturing complexity and introduce an additional wick layer.

Mesh and composite-mesh wicks can improve liquid transport through multiscale pores. The capillary factors reported for copper woven mesh wicks [31], multilayer copper mesh [32], copper spiral woven mesh (SWM) [33], and 2D/3D composite wicks [34] are 10.84 mm·s^−0.5^, 8.85 mm·s^−0.5^, 14.20 mm·s^−0.5^ and 19.44 mm·s^−0.5^, respectively. Although these structures provide good pore connectivity, they generally require weaving, stacking, or composite assembly and occupy part of the limited internal space in ultrathin two-phase heat-transfer devices. Hydrophilic nylon-mesh wicks [35] exhibit a capillary factor of 9.36 mm·s^−0.5^ and offer advantages in flexibility and lightweight construction for flexible thermal-management applications, although their capillary transport performance is comparatively lower.

Regular microgrooves provide continuous, directional liquid-transport pathways. Multiscale ceramic microgroove wicks have achieved a capillary factor of 21.55 mm·s^−0.5^ [19]. In this study, MOPA nanosecond-laser texturing directly formed interconnected main microgrooves, recast ridges, and secondary microgrooves on 316L stainless steel. Under the preferred condition of 15 scanning passes, 0.1 mm groove spacing, and 200 ns pulse width, the capillary factor reached 30.859 mm·s^−0.5^, the average rise velocity over the first 10 s reached 8.57 mm·s^−1^, and the liquid front reached the 150 mm measurement limit in approximately 35 s. The capillary factor exceeded the values listed in Table 4, although differences in materials and testing conditions limit direct comparisons. The observed performance is consistent with the main grooves providing liquid-transport space and the connected secondary grooves offering auxiliary capillary pathways. Direct texturing provides a potential route to integrated shell–wick fabrication for ultrathin stainless-steel vapor chambers without separate wick layers.

## 4. Conclusions

Hierarchical main–secondary microgroove wicks were fabricated on 316L stainless steel using MOPA nanosecond-laser texturing. The effects of processing parameters on groove morphology, connectivity, and capillary transport were investigated. The main conclusions are as follows:

(1) Recast ridges with local basin-like openings connected the secondary microgrooves to the main channels. Excessive scanning passes or pulse widths produced continuous wall-like ridges that narrowed channel openings and weakened connectivity. Small groove spacings caused neighboring recast structures to merge, whereas no identifiable open secondary microgrooves were observed at excessively large spacings.
(2) The preferred condition was 15 scanning passes, a groove spacing of 0.1 mm, and a pulse width of 200 ns. The main microgrooves were 60 μm wide and 117.1 μm deep, measured from the ridge apex to the groove bottom, with a recast-ridge height of 49.4 μm above the original surface. The secondary microgrooves were 52 μm wide and 38.8 μm deep. The initial apparent water contact angle decreased from 84.5° to 18.9°, with complete wetting observed at 0.32 s. The capillary factor reached 30.859 mm·s^−0.5^, the average rise velocity over the first 10 s reached 8.57 mm·s^−1^, and the liquid front reached the 150 mm measurement limit in approximately 35 s.
(3) The results support the expectation that effective main–secondary connectivity improves capillary transport, while further secondary-microgroove development alone does not necessarily improve performance. Four independently fabricated specimens exhibited similar capillary-rise curves under the preferred condition, supporting specimen-to-specimen reproducibility. This approach provides a potential route to integrated shell–wick fabrication for ultrathin stainless-steel vapor chambers without additional mesh or sintered-powder layers.

## Figures and Tables

**Table 1:** Chemical composition of 316L stainless steel.

Chemical Composition (%)	C	Si	Mn	P	S	Ni	Cr	Mo	Fe
	0.015	0.409	1.190	0.031	0.0016	10.040	16.910	2.006	Bal.

**Table 2:** Sample processing parameters.

Sample	Dimension (mm)	Passes	Spacing (mm)	Pulse Width (ns)
Untreated	1.5 × 150	0	0	0
CJ5	1.5 × 150	5	0.1	200
CJ10	1.5 × 150	10	0.1	200
CJ15	1.5 × 150	15	0.1	200
CJ20	1.5 × 150	20	0.1	200
CJ25	1.5 × 150	25	0.1	200
CJS0.05	1.5 × 150	15	0.05	200
CJS0.1	1.5 × 150	15	0.1	200
CJS0.2	1.5 × 150	15	0.2	200
CJS0.3	1.5 × 150	15	0.3	200
CJS0.4	1.5 × 150	15	0.4	200
CJPW50	1.5 × 150	15	0.1	50
CJPW100	1.5 × 150	15	0.1	100
CJPW200	1.5 × 150	15	0.1	200
CJPW300	1.5 × 150	15	0.1	300
CJPW400	1.5 × 150	15	0.1	400

**Table 3:** 3D profile parameters of the samples.

Sample	Main Microgroove Depth (μm)	Main Groove Depth from Reference Surface (μm)	Main Microgroove Width (μm)	Secondary Microgroove Depth (μm)	Secondary Microgroove Width (μm)
Untreated	0	0	0	0	0
CJ5	72.8	41.8	70	20	43
CJ10	89	49.2	62	30	43
CJ15	117.1	67.7	60	38.8	52
CJ20	131.7	70.7	58	49.6	51
CJ25	138.7	79.9	55	54.1	62
CJS0.05	67.4	44.9	48	**-**	**-**
CJS0.1	117.1	67.7	60	38.8	52
CJS0.2	91.8	50	71.7	**-**	**-**
CJS0.3	94.7	52.8	75.9	**-**	**-**
CJS0.4	94.4	50.7	85	**-**	**-**
CJPW50	79.2	43.7	54	-	-
CJPW100	111.4	52.9	57.3	-	-
CJPW200	117.1	67.7	60	38.8	52
CJPW300	111.2	71.1	54	54.6	59
CJPW400	126.6	73.7	54.4	52.4	64.1

**Table 4:** Comparison of the capillary performance of laser-textured 316L stainless steel hierarchical microgroove wicks with previously reported wicks.

Literature	Wick Structure	Working Fluid	Maximum Capillary Rise Height (mm)	Capillary Factor (mm·s^−0.5^)
**This study**	**316L stainless steel**	**Deionized water**	**150**	**30.859**
Yuan et al. [19]	aluminium oxide	Deionized water	114	21.55
Li et al. [29]	Copper powder sintered	Deionized water	55	14.65
Li et al. [30]	316L stainless-steel gradient sintered-powder wick	Deionized water	~42	4.5
Wu et al. [31]	Copper woven mesh	Deionized water	80.6	10.84
Chen et al. [32]	Multilayer copper mesh	Deionized water	55.98	8.85
Chen et al. [33]	Copper SWM	Deionized water	~75	14.2
Yan et al. [34]	2D/3D composite wick	Deionized water	69.4	19.44
Hu et al. [35]	Nylon mesh	Deionized water	81	9.36

## Data Availability

The original contributions presented in this study are included in the article/Supplementary Material. Further inquiries can be directed to the corresponding author.

## Supplementary Materials

The following supporting information can be downloaded at: https://www.mdpi.com/article/10.3390/ma19214619/s1, Figure S1: EDS spectrum of the untreated 316L stainless-steel surface; Figure S2: EDS spectrum of the laser-textured 316L stainless-steel surface under the preferred processing condition; Figure S3. Selected EDS X-ray intensity maps of the untreated reference (a–e) and laser-textured specimen (f–j): C, N, O, S, and Zn, respectively. The maps are presented qualitatively and are not used to compare elemental concentrations across different elements.; Table S1: Reported EDS elemental compositions of the untreated and laser-textured specimens.
