# Supplementary material for: Tunable Capillary Wicking of 316L Stainless-Steel Hierarchical Microgrooves Fabricated by Master Oscillator Power Amplifier Nanosecond Laser Texturing
Source: Materials (Basel). 2026 Oct 30;19(21):4619. doi: 10.3390/ma19214619 (PMC13765200; doi:10.3390/ma19214619)
Supplement: Supplementary file 1 [file materials-19-04619-s001.zip › materials-4638448-supplementary.pdf]

Tunable capillary wicking of 316L stainless-steel hierarchical microgrooves fabricated by master oscillator power amplifier nanosecond laser texturing

Supplementary material S1: EDS characterization of untreated and laser-textured 316L stainless steel

Energy-dispersive X-ray spectroscopy (EDS) was performed on the untreated 316L stainless-steel reference and the laser-textured specimen prepared under the preferred condition of 15 scanning passes, a groove spacing of 0.1 mm, and a pulse width of 200 ns. Representative spectra, selected X-ray intensity maps, and reported elemental compositions are presented in Figures S1–S3 and Table S1.

Both specimens exhibited Fe, Cr, Ni, and Mo signals. The EDS-reported Fe mass fraction was 64.48 wt% for the untreated reference and 64.94 wt% for the laser-textured specimen. The corresponding carbon mass fractions were 6.19 and 4.17 wt%, while the chromium mass fractions were 16.08 and 18.39 wt%, respectively. These results describe the reported elemental composition of the examined regions but do not independently determine surface chemical states or surface free energy.

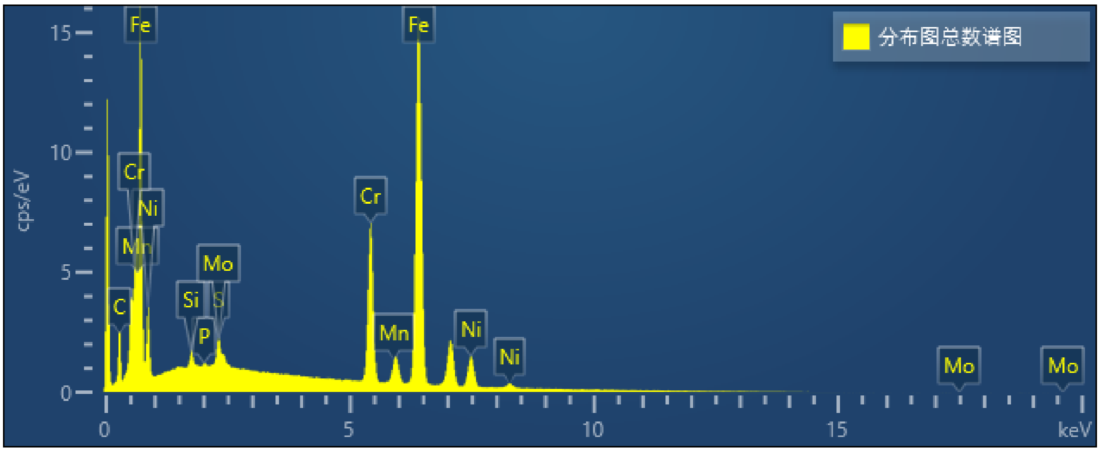

Figure S1. EDS spectrum of the untreated 316L stainless-steel surface.

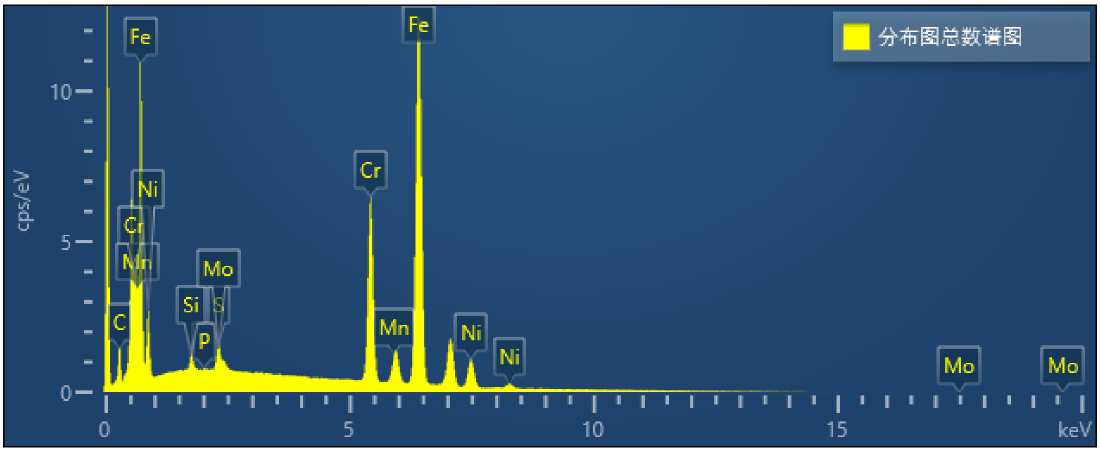

Figure S2. EDS spectrum of the laser-textured 316L stainless-steel surface under the preferred processing condition.

Table S1. Reported EDS elemental compositions of the untreated and laser-textured specimens.

Element	Untreated, wt%	Laser-textured, wt%	Untreated, at%	Laser-textured, at%
C	6.19	4.17	23.41	16.73
Si	0.43	0.40	0.70	0.69
P	0.00	0.00	0.00	0.00
S	0.00	0.00	0.00	0.00
Cr	16.08	18.39	14.04	17.03
Mn	1.34	1.74	1.11	1.53
Fe	64.48	64.94	52.42	56.01
Ni	9.67	8.81	7.48	7.23
Mo	1.79	1.55	0.85	0.78

Values are reproduced from the EDS quantification outputs. Small deviations of the summed values from 100% arise from rounding. N, O, and Zn were not listed in these quantitative outputs. Values of 0.00 do not establish the absolute absence of an element.

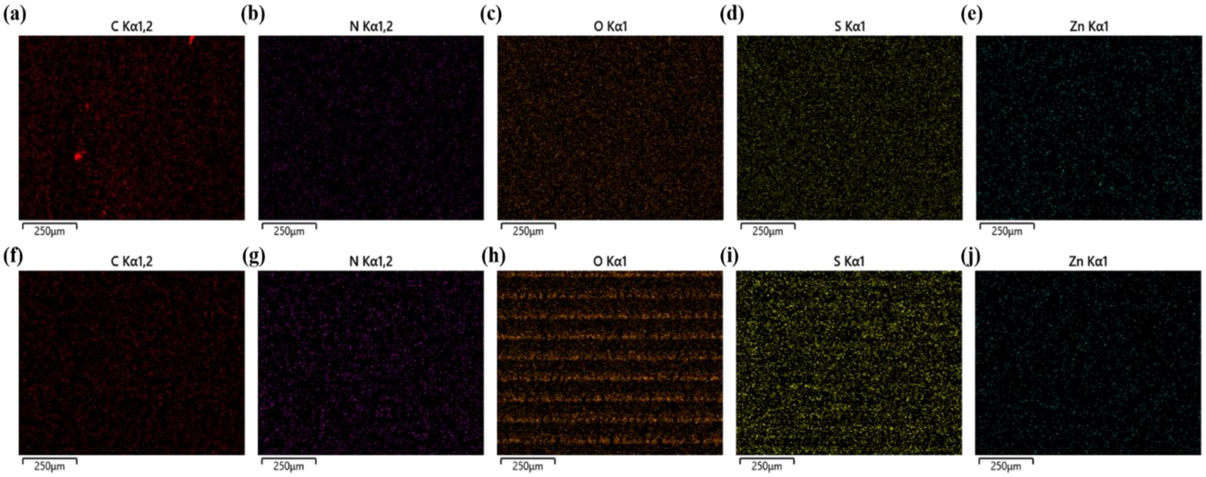

Figure S3. Selected EDS X-ray intensity maps of the untreated reference (a–e) and laser-textured specimen (f–j): C, N, O, S, and Zn, respectively. The maps are presented qualitatively and are not used to compare elemental concentrations across different elements.
